# Actinomycosis on the Finger, Taken from a Calf

**Published:** 1897-04

**Authors:** J. R. Snively

**Affiliations:** Lanark, Ill.


### ACTINOMYCOSIS ON THE FINGER, TAKEN FROM A CALF.

By J. R. Snively, V.S.,
LANARK, ILL.

On October 22d last I was called to see a case of actinomycosis in a four-months-old calf. In catching the calf I was unfortunate enough to have quite a large piece of the skin knocked off my right forefinger. I opened the abscess and treated it; afterward washed my hands in a 3 per cent. solution of creolin, but the germ must have found entrance. The wound seemed to heal nicely for about ten days, when two small, red-looking tumors began growing. I cauterized these with carbolic acid, which seemed to have no effect, and they kept growing. I then cauterized them with antimony chloride and at the same time took twice daily 5 grains iodide of potash with good results. About one month after they had healed they commenced to itch and raise up again. This time I used 10 grains twice a day of the iodide of potash, and have not been troubled with them since.

